# ECM proteins regulate microRNA-mediated direct reprogramming of fibroblasts into cardiomyocytes via YAP signaling

**DOI:** 10.3389/fbioe.2026.1749865

**Published:** 2026-03-12

**Authors:** Gerardina Ruocco, Letizia Nicoletti, Martina Coletto, Alessia Toccaceli, Valeria Chiono, Camilla Paoletti

**Affiliations:** 1 Department of Mechanical and Aerospace Engineering (DIMEAS), Politecnico di Torino, Torino, Italy; 2 The Interuniversity Center for the Promotion of the 3Rs Principles in Teaching and Research, Centro 3R, Torino, Italy; 3 PoliRNA srl, Torino, Italy; 4 Department of Physiological Sciences, Faculty of Medicine and Health Science, University of Barcelona, Barcelona, Spain; 5 Laboratory of Cancer Metabolism, ONCOBELL Program, Bellvitge Biomedical Research Institute (IDIBELL), L’Hospitalet de Llobregat, Barcelona, Spain; 6 Institute for Chemical and Physical Processes (IPCF), National Research Council (CNR), Pisa, Italy

**Keywords:** DE-DOPE, ECM cardiac proteins, fibroblast reprogramming, induced cardiomyocytes, miRcombo, YAP signaling

## Abstract

**Introduction:**

Direct cardiac reprogramming represents a promising strategy to regenerate damaged myocardium by converting cardiac fibroblasts into induced cardiomyocytes (iCMs). Transient delivery of a four-microRNA cocktail (miRcombo: miR-1, miR-133, miR-208, and miR-499) has been shown to activate cardiac transcriptional programs in adult human cardiac fibroblasts (AHCFs). However, *in vitro* reprogramming efficiency remains limited compared to significantly higher outcomes observed in vivo, suggesting that microenvironmental cues present in the native myocardium play a crucial role in facilitating lineage conversion. This study investigated how extracellular matrix (ECM) proteins modulate miRcombo-mediated reprogramming.

**Methods:**

An *in vitro* cardiac ECM termed “biomatrix” was developed and characterized from long-term cultured AHCFs. An optimized decellularization protocol was applied to preserve major ECM components, including laminin, fibronectin, and collagen type I, while minimizing residual DNA content. Lipoplexes composed of [2-(2,3-didodecyloxypropyl)-hydroxyethyl] ammonium bromide (DE) and dioleoyl phosphatidylethanolamine (DOPE) were used to transiently transfect AHCFs with miRcombo. Cells were cultured on coatings of individual ECM proteins (laminin, fibronectin, collagen I) or biomatrix to assess the influence of cell–substrate interactions on reprogramming efficiency. Analyses were conducted at 7 and 15 days post-transfection.

**Results:**

Biomatrix significantly enhanced reprogramming efficiency, yielding approximately 20% cardiac Troponin T (cTnT)^+^ cells compared to other substrates. Gene expression analyses demonstrated marked upregulation of cardiac markers TNNT2, ACTC1, and CACNA1C in biomatrix-cultured cells. Structural assessment revealed improved cytoskeletal alignment and sarcomeric organization on laminin and biomatrix, whereas fibronectin and collagen I supported poorer structural maturation. At 3 days post-seeding, fibronectin and collagen I promoted higher proliferation rates and increased nuclear localization of YAP, while laminin and biomatrix reduced YAP activation, favoring cardiac transdifferentiation over proliferation.

**Discussion:**

These findings demonstrate that ECM biochemical cues are key regulators of direct cardiac reprogramming. Laminin- and biomatrix-enriched microenvironments enhance miRcombo-mediated iCM induction efficiency *in vitro*, potentially by modulating YAP signaling and balancing proliferation versus transdifferentiation. This study highlights the importance of recapitulating native cardiac microenvironmental signals to improve the efficacy of direct cardiac reprogramming strategies.

## Graphical Abstract

**Figure:**
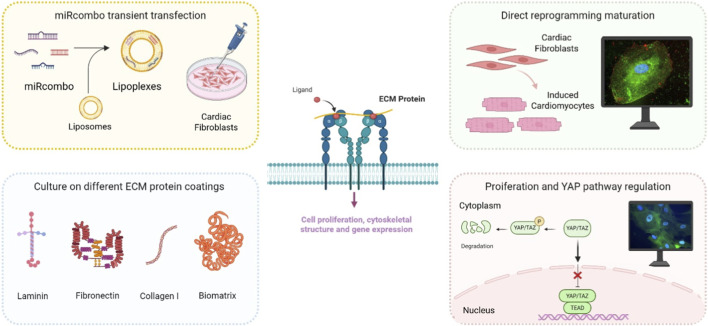

## 1 Introduction

Direct reprogramming enables the trans-differentiation of endogenous fibroblasts into specialized cells by activating lineage-specific transcriptional pathways without passing through a pluripotent state. This approach has attracted increasing interest for regenerative applications, disease modeling, and screening platforms (Srivastava and DeWitt, 2016; Isomi et al., 2019). In cardiac regenerative medicine, direct reprogramming can be exploited to generate induced cardiomyocytes (iCMs), offering a strategy to simultaneously reverse fibrosis and replenish contractile cells in diseased conditions. Specifically, the direct reprogramming of scar fibroblasts into iCMs has emerged as a promising approach to restore cardiac function after myocardial infarction (MI) (Wang et al., 2024). Early direct reprogramming strategies relied on the overexpression of cardiac transcription factors such as Gata4, Mef2C, and Tbx5 (GMT), or GMT together with Hand2 (GHMT), which induced the formation of iCMs (Ieda et al., 2010; Song et al., 2012). When delivered *in vivo* following MI, GMT or GHMT expression were able to reprogram resident cardiac fibroblasts (CFs), leading to reduced scar formation and improved cardiac function (Qian et al., 2012; Inagawa et al., 2012; Miyamoto et al., 2018). More recently, a microRNA-based approach has been established using a combination of four microRNAs, miR-1, miR-133, miR-208, and miR-499 (miRcombo), which modulate post-transcriptional gene regulation. miRcombo is able to suppress fibroblast-associated programs while promoting cardiac lineage gene expression, leading to the generation of iCMs-like cells in both murine and adult human cardiac fibroblasts (AHCFs) (Wang et al., 2024; Jayawardena et al., 2012; Jayawardena et al., 2014; Hu et al., 2021; Sun et al., 2023; Paoletti et al., 2020; Nicoletti et al., 2022; Visone et al., 2024; Nicoletti et al., 2025). However, despite significant progress, reprogramming efficiency and iCMs maturation *in vitro* remain limited, suggesting that additional cues beyond reprogramming factors, such as biophysical and extracellular microenvironmental factors, play a key role in directing cell fate conversion (Paoletti and Chiono, 2021; Paoletti et al., 2022). Indeed, directly reprogrammed cells obtained *in vivo* display a more mature and functional cardiomyocyte-like phenotype compared to those obtained in standard *in vitro* cultures (Jayawardena et al., 2014). This difference points out the importance of the microenvironment, which provides biochemical stimuli, three dimensional (3D) architectural organization and physiological stiffness which are not reproduced on rigid polystyrene culture surfaces (Srivastava and DeWitt, 2016; Yamakawa and Ieda, 2021; Spedicati et al., 2024; Spedicati et al., 2022; Ruocco et al., 2023; Li Y. et al., 2016).

The extracellular matrix (ECM) is a major component of cardiac microenvironment, providing biochemical and biophysical cues that influence cell adhesion, cytoskeletal organization, and gene expression (Paoletti and Chiono, 2021). ECM properties such as protein composition and substrate stiffness are recognized by cells and transmitted to the nucleus through mechanotransduction pathways (Crowder et al., 2016; Dupont, 2016). In the myocardium, ECM components such as laminin, fibronectin, and collagen I form a highly specialized 3D network that influences cardiomyocyte phenotype, alignment, and electrophysiological properties (Paoletti et al., 2022; Li et al., 2024).

In our previous work we have already demonstrated that after 2 weeks from miRcombo-mediated transfection, early cardiac transcription factors such as Gata4, Mef2C, Tbx5, Hand2, and Nkx2.5 were significantly upregulated in the AHCFs cultured onto cell-derived biomatrix, demonstrating that biochemical and biophysical stimuli improved direct reprogramming outcomes (Paoletti et al., 2022). Moreover, early during reprogramming, fibroblasts have been shown to upregulate cardiac ECM proteins such as collagens and laminins, indicating that cells actively attempt to reconfigure their microenvironment to facilitate fate transition (Paoletti and Chiono, 2021). Smith *et al.* developed a polyethylene glycol (PEG)-based hydrogel substrates functionalized with high concentrations of laminin and Arginine-Glycine-Aspartic acid (RGD) adhesion peptides, achieving significantly enhanced fibroblast reprogramming into iCMs compared to hydrogels with lower adhesion motif densities or standard tissue culture polystyrene (Smith et al., 2013). These findings suggest that the efficiency of cardiac reprogramming is strongly governed by ECM-derived cues, acting through mechanical and biochemical cues that influence cell behavior, signaling and lineage-specific gene expression.

The Hippo signaling pathway regulates cell proliferation, cell fate decisions, and cellular plasticity through a kinase cascade that controls the activity of the transcriptional co-activators Yes-associated protein (YAP) and transcriptional co-activator with PDZ-binding motif (TAZ). When the Hippo pathway is active, YAP is phosphorylated and retained in the cytoplasm, thereby limiting its transcriptional activity. When the pathway is inactive, YAP becomes dephosphorylated and translocate to the nucleus, where it interacts with TEAD transcription factors to regulate gene expression programs associated with proliferation and differentiation (Dupont, 2016; Di Benedetto et al., 2021). YAP and TAZ also act as mechanosensitive transcriptional regulators whose nuclear-cytoplasmic localization is influenced by biomechanical and microenvironmental cues, including ECM composition, substrate stiffness, cell density, and cytoskeletal tension. Through this role, they translate extracellular physical signals into transcriptional responses that modulate cell fate and cellular plasticity (Dupont, 2016; Tschumperlin et al., 2018). Consistently, recent studies have shown that mechanical properties of the microenvironment modulate direct reprogramming efficiency also via YAP/TAZ signaling. Softer matrices, more similar to native myocardium promote reprogramming by inhibiting YAP/TAZ activity and suppressing fibroblasts gene programs (Kurotsu et al., 2020). More broadly, Hippo/YAP pathway modulation have an impact on cell plasticity and cellular reprogramming (Di Benedetto et al., 2021; Moya and Halder, 2016).

In this work, we aimed to investigate how distinct ECM components (laminin, fibronectin and collagen I) and a cardiac tissue–like biomatrix (BM) derived from long term *in vitro* culture of AHCFs influenced fibroblast reprogramming. Lipoplexes based on cationic lipid [2-(2,3-didodecyloxypropyl)-hydroxyethyl] ammonium bromide (DE) and helper lipid dioleoyl phosphatidylethanolamine (DOPE) were used for miRcombo delivery to AHCFs (DE-DOPE/miRcombo), as this formulation has been previously shown to ensure efficient microRNA encapsulation, enhance cellular uptake and cell viability, and support AHCFs reprogramming into iCMs better compared to commercial transfection agent DharmaFECT1 (Nicoletti et al., 2022). We developed an optimized decellularization protocol to obtain an ECM-rich biomatrix preserving the key structural proteins of the cardiac niche. We then compared the efficiency of fibroblast reprogramming on single ECM coatings laminin (LN), fibronectin (FN), and collagen I (CI) and on BM substrate. Finally, we assessed YAP signaling activity through drug-mediated blocking in relation to the different coatings and miRcombo treatment to better understand its association with reprogramming efficiency.

## 2 Materials and methods

### 2.1 Culture of adult human cardiac fibroblasts (AHCFs)

Adult human cardiac fibroblasts (AHCFs) were purchased from Lonza (CC-2903) (Walkersville, MD, USA) and were maintained in Dulbecco’s modified Eagle medium high glucose (DMEM, Gibco), 10% fetal bovine serum (FBS, Sigma-Aldrich), 1% L-glutamine (Sigma-Aldrich) and 1% penicillin-streptomycin (Sigma-Aldrich). Cells were expanded until passage four and then used for experiments.

### 2.2 AHCF-derived biomatrix (BM) production *in vitro*

AHCFs (5 × 10^5^ cells/dish) were cultured in 100 mm-diameter Petri in Fibroblast Growth Medium-3 (FGM-3, Lonza, CC-4526) for up to 21 days, allowing BM deposition. Decellularization was performed by incubation in 0.25% Triton X-100 (Sigma-Aldrich) and 10 mM NH_4_OH (Sigma-Aldrich) in phosphate buffer saline (PBS Gibco, Waltham, MA, USA) prewarmed to 37 °C, for 2 min. After twice washed in ultrapure H_2_O, BM was collected in 1 mL of ultrapure H_2_O and freeze-dried (CoolSafe 4–15 L freeze-dryer, Labogene, Scandinavia) overnight. BM powder was stored at −20 °C for experiments.

### 2.3 DNA quantification after decellularization

AHCFs (4 × 10^4^ cells/dish) were plated in 12-multiwell in Fibroblast Growth Medium-3 (FGM-3, Lonza, CC-4526) for up to 21 days and decellularized. Non-decellularized samples were used as controls. Decellularized and control cell samples were resuspended in Reaction Buffer (10 mM Tris-HCl, 2.5 mM MgCl_2_, 0.5 mM CaCl_2_, pH 7.5). When indicated, samples were treated with DNase I (1 U/µL, Thermo Scientific™) and incubated for 30 min at 37 °C to degrade potential remaining DNA. A 1.5% (w/v) agarose gel was prepared in 1× TAE buffer and stained with SYBR Safe™ DNA Gel Stain (1:100000 dilution). Samples (5–10 µL) were mixed with 6× loading dye and loaded alongside a 100 bp DNA ladder. Electrophoresis was performed at 150 V for 15 min using a horizontal gel system. Gels were imaged using a ChemiDoc™ Imaging System (Bio-Rad), and DNA retention was evaluated based on band visibility and intensity. Residual DNA content was then quantified by UV-Vis spectrophotometry. Lyophilized BM samples were resuspended after decellularization (± DNase I treatment as described) and measured in microvolume mode using NanoQuant plate (Tecan Group Ltd. Männedorf, Switzerland).

### 2.4 BM protein quantification

AHCFs (4 × 10^4^ cells/dish) were plated in 12-multiwell in Fibroblast Growth Medium-3 (FGM-3, Lonza, CC-4526) for up to 21 days and decellularized. Non-decellularized samples were used as controls. Decellularized and control cell samples were treated with 100 µL of RIPA buffer (Cell Signaling) following manufacturer’s instructions. Total protein content in BM was quantified using a bicinchoninic acid (BCA) assay (Pierce™ BCA Protein Assay Kit, Thermo Scientific) following the microplate procedure. A BSA standard curve (0–2000 μg/mL) was prepared by serial dilution in PBS. Samples and standards (25 µL per well, in triplicate) were dispensed into a 96-well plate. Working Reagent (WR) was prepared fresh by mixing Reagent A with Reagent B at 50:1 (v/v) and 200 µL of WR were added to each well. Plates were sealed and incubated at 37 °C for 30 min (protected from light), then cooled to room temperature. Absorbance was read at 562 nm with a microplate reader (VarioskanTM LUX multimode). Protein concentrations were obtained from the BSA calibration curve (linear regression within the assay’s linear range). Results were expressed as µg/mL of protein.

### 2.5 Scanning electron microscopy of BM

AHCFs were seeded at a density of 2.2 × 10^4^ cells per well on autoclaved glass coverslips placed in 24-well plates and cultured in FGM-3 for 21 days to allow BM deposition. At the end of the culture period, samples were either decellularized as described above or processed as cellularized matrices. All samples were fixed in 4% paraformaldehyde (PFA) for 10 min at room temperature and washed three times with PBS. Samples were dehydrated through washes in serial dilution of ethanol (30%, 50%, 70%, 80%, 90%, 96%, 100%, and 100%), with each step lasting 5 min. After dehydration, coverslips were mounted on stubs and sputter-coated with gold for 70 s using an AGB7234 high-resolution sputter coater. SEM imaging of BM was performed using a TESCAN VEGA scanning electron microscope (TESCAN Orsay Holdings, Brno, Czech Republic) operated in resolution scan mode with an accelerating voltage of 10 kV and a beam current of 1 nA. The magnifications were set at ×250 and ×500.

### 2.6 BM immunofluorescence

AHCFs (3 × 10^4^ cells/dish) were plated on 35 mm µ-Dishes (Ibidi, Gräfelfing, Germany) and cultured for 21 days as described before. To characterize BM after decellularization, samples were decellularized as previously described, fixed in 4% paraformaldehyde (PFA, Alfa Aesar, Ward Hill, MA, USA) and blocked with bovine serum albumin (BSA) 1% in PBS for 1 h. Samples were then incubated with primary antibodies against laminin (L827-1 Sigma-Aldrich), fibronectin (F3648 Sigma-Aldrich), collagen I (C2456 Sigma-Aldrich), overnight at 4 °C. After washing with PBS, samples were stained with Alexa-488 (120077 Invitrogen) and 555 secondary antibodies (A21422 Invitrogen), for 2 h at room temperature. Nuclei were counterstained with 40,6-diamidino-2-phenylindole (DAPI, Invitrogen). Images were acquired using the Spinning Disk Ti-2 Eclipse microscope (Nikon, Tokyo, Japan) at ×20 magnification. Five randomly chosen microscopic fields were taken for each sample. Experiments were performed in biological and technical triplicates.

### 2.7 Cell transfection with microRNAs and plating on ECM protein coatings

AHCFs were seeded in 6-well plates (1.1 × 10^5^ cells/well) in DMEM, with 10% FBS and 1% glutamine. After 24 h from seeding, AHCFs were transfected with miRcombo (miR-1-3p, miR-133a-3p, miR-208a-3p, and miR-499a-5p, mirVana® miRNA mimic, Life Technologies) loaded into DE-DOPE liposomes (([2-(2,3-didodecyloxypropyl)-hydroxyethyl] ammonium bromide, DE, Nanosoft and Lalpha-dioleoyl phosphatidylethanolamine, DOPE, Sigma-Aldrich), to form DE-DOPE lipoplexes, as described by Nicoletti et al. (2022); Nicoletti et al. (2025). Briefly, empty DE-DOPE liposomes were prepared using the thin lipid film-hydration method (D’Souza, 2017). The dried film of DE-DOPE was dissolved in RNase-free water (Fisher Bioreagents) to a final concentration of 1 mg mL−1 of the liposomal suspension. Then, DE-DOPE/miRcombo lipoplexes were prepared at amino to phosphate groups (N/P) ratio of 3, as previously optimized (Nicoletti et al., 2022), by mixing 10 µL of miRcombo solution (5 µM) and 6 µL of DE-DOPE suspension. DE-DOPE/miRcombo lipoplexes were incubated for 20 min at room temperature, vortexed, and then incubated for additional 20 min. DE-DOPE/miRcombo lipoplexes were administered to AHCFs at a final concentration of 25 nmol L−1 in DMEM High Glucose supplemented with 10% FBS and 1% glutamine. After 24 h of DE-DOPE/miRcombo lipoplexes treatment, cells were trypsinized and then seeded onto ECM-protein coated 24-well plates (22 × 10^3^ cells/well) for flow cytometry and droplet digital PCR (ddPCR) analysis, or 96-well plates (5 × 10^3^ cells/well) for immunofluorescence. ECM-protein coatings were prepared as follow: 50 μg/mL of sterile-filtered protein solutions of human recombinant laminin 211 (BioLamina), fibronectin (Sigma-Aldrich), human collagen type I (Corning) and BM were prepared prior to cell seeding. Specifically, BM solution was obtained by dissolving BM powder in PBS, without stirring at 37 °C, until complete dissolution. Coating solutions were incubated for 2 h at 37 °C. Finally, the excess was removed, and the coated surfaces were washed with HBSS, prior to cell culture. Uncoated polystyrene (PS) dishes were used as control. For the YAP blocking experiments, cells were transfected with miRcombo or negmiR (Negative Control #1, mirVana™ miRNA Mimic, Life Technologies) using DEDOPE in DMEM high glucose supplemented with 10% FBS and 1% glutamine. After transfection with DE-DOPE/miRcombo or DE-DOPE/negmiR, cells were re-plated onto different ECM protein coatings and treated with the ROCK inhibitor Y-27623 (Sigma-Aldrich) at 30 µM for 15 days in the same culture medium (Kurotsu et al., 2020). The culture medium was replaced every other day with freshly prepared medium supplemented with ROCK inhibitor.

### 2.8 Immunofluorescence

AHCFs at different stages of reprogramming (3,7 and 15 days post-seeding on ECM protein coatings) were fixed and treated for immunofluorescence staining in order qualitatively assessed the expression Ki67 proliferation marker, YAP transcriptional co-factor, Mef2C cardiac transcriptional factor, cardiac troponin T (cTnT) and alpha-sarcomeric actinin (a-SAR). At selected time point, AHCFs were fixed in 4% PFA (Alfa Aesar), permeabilized 0.01% Triton X 100 (Sigma-Aldrich) for 10 min, and blocked with BSA 1% in PBS for 1 h. Cells were incubated with primary antibodies against Ki67 (UM870033, UltraMAB), YAP (ab52771, abcam), Mef2C (5030, Cell Signaling), cTnT (701620, Invitrogen) and α-SAR (A7607 Sigma-Aldrich) overnight at 4 °C. After washing with PBS, samples were stained with Alexa-647 (Invitrogen) and Alexa-488 (Invitrogen) secondary antibodies for 2 h at room temperature. Cells incubated only with secondary antibody were used as negative control. Nuclei were counterstained with DAPI, while the cytoskeleton was counterstained with Phalloidin (Rhodamine Phalloidin F-Actin, Thermo Fisher Scientific). Images were acquired using Spinning Disk Ti-2 Elipse microscope (Nikon) at ×20 and ×60 magnification and merged using ImageJ software. The percentage of Ki67, YAP, Mef2C and cTnT-positive cells was estimated by counting the number of cTnT-positive cells with respect to the total number of cells. YAP subcellular distribution was quantified from immunofluorescence images according to Li et al. protocol. Image analysis was performed in ImageJ/Fiji. Nuclei were segmented using the DAPI channel and a nuclear region of interest (ROI) was generated for each cell. The corresponding cytoplasmic ROI was defined by subtracting the nuclear ROI from the whole-cell area, which was manually selected based on YAP signal to avoid including background. For each cell, the mean fluorescence intensity of YAP within the nuclear ROI and cytoplasmic ROI was measured after background subtraction. The nuclear-to-cytoplasmic ratio (N/C) of YAP intensity was calculated as:

$$YAPratio\left(\frac{N}{C}\right)=\frac{I_{\text{nucleus}}}{I_{\text{cytoplasm}}}$$

where $I_{\text{nucleus}}$ and represent background-corrected mean intensities in the nuclear and cytoplasmic ROIs, respectively. Both the percentage of nuclear YAP-positive cells and the YAP N/C ratio were plotted. At least 20 cells per condition were quantified across 3 independent experiments.

### 2.9 Flow cytometry

Flow cytometry analysis to assess reprogramming efficiency was performed 15 days post-transfection. AHCFs were seeded at a density of 22,000 cells/well onto 24-well plates pre-coated with the respective ECM substrates. Cells were detached using 0.05% Trypsin/EDTA (Sigma-Aldrich) for 5 min at 37 °C. After centrifugation (1800 rpm, 5 min), cells were washed in ice-cold PBS supplemented with 10% FBS. Ice-cold PBS with 10% FBS was used for all the following washes. Cells were permeabilized with 0.5% (v/v) Tween-20 in PBS and incubated on ice for 10 min. For staining, cells were incubated with anti-cardiac Troponin T (cTnT) primary antibody (701620, Invitrogen) for 1 h at 4 °C and Alexa Fluor 488-conjugated secondary antibody (Abcam) for 30 min at 4 °C in the dark. Cells were analyzed using a Guava EasyCyte Flow Cytometer (Merck, Kenilworth, NJ, USA). Data were processed using GuavaSoft 3.2, and gating was performed based on unstained and secondary-only controls.

### 2.10 RNA isolation and ddPCR

After 3 and 15-day post cell seeding on ECM protein coatings, total RNA was extracted using QIAzol Lysis Reagent (Qiagen, Hilden, Germany), according to the manufacturer’s instructions. RNA quality and concentration were evaluated using a NanoQuant plate (Tecan Group Ltd., Männedorf, Switzerland). cDNAs were obtained using the High-Capacity cDNA Reverse Transcription Kit (Applied Biosystems, Waltham, MA, USA). Gene expression was evaluated by droplet digital PCR (ddPCR) (Bio-Rad Laboratories, Hercules, CA, USA), allowing the quantification of few amounts of template and able to discriminate with high precision very small differences in gene expression. Specifically, the following genes were examined: YAP1 (ID assay: dHsaCPE5030690), WWTR1, TAZ (ID assay: dHsaCPE5191727), TEAD1 (ID assay: dHsaCPE5042644), cardiac troponin T2 (TNNT2, ID assay: dHsaCPE5052344), calcium voltage-gated channel subunit alpha1 C (CACNAC1, ID assay: dHsaCPE5038360) and actin alpha cardiac muscle 1 (ACTC1, ID assay: qHsaCIP0028009).

To prepare for the experiment, the primers and probes (Bio-rad Laboratories) labeled with carboxyfluorescein (FAM)/hexachlorofluorescein (HEX) were mixed with 10 μL of ddPCR Super-mix for probes (no dUTP), 5 μL of 20 ng cDNA and 4 μL H_2_O up to a final volume of 20 μL/reaction. This mixture was then added to the Bio-Rad DG8 disposable droplet generator cartridge, which was filled with 50 μL of droplet generation oil into the oil well for each sample. The cartridge was placed into the QX100 droplet generator (Bio-Rad) and droplet generation was conducted following the manufacturer’s instructions. The resulting droplets were transferred to a ddPCRTM 96-well PCR plate (Bio-Rad) and placed in a T100 thermal cycler (Bio-Rad). Thermal cycling conditions were 95 °C for 10 min (1 cycle), 94 °C for 30 s and 55 °C for 30 s (40 cycles), 98 °C for 10 min (1 cycle), and 4 °C infinite hold. Then, the droplets were analyzed for DNA copy number quantification by loading the PCR plate onto a BioRad QX100 droplet reader. The resulting data was then analyzed using the QuantaSoft analysis software (Bio-Rad Laboratories). To perform quantitative normalization, the housekeeping gene Glyceraldehyde 3-phosphate dehydrogenase (GAPDH; ID assay: dHsaC-PE5031597) was used. The results were reported as the concentration (cDNA copies/μL) of the gene of interest normalized on the concentration mean (cDNA copies/μL) of GAPDH. No template control with water was included in each assay. Data are presented as mean ± SEM. Experiments were performed in triplicate.

### 2.11 Statistical analysis

The results are shown as means ± standard error of the mean (SEM). All experiments were performed in triplicate. Statistical analyses were carried out with ANOVA test, with p-value reported as *p < 0.05 considered statistically significant, **p < 0.01 considered highly significant and ***p < 0.0001 very highly significant. All graphs were prepared using GraphPad software.

## 3 Results

### 3.1 Biomatrix optimization and characterization

**FIGURE 1:**
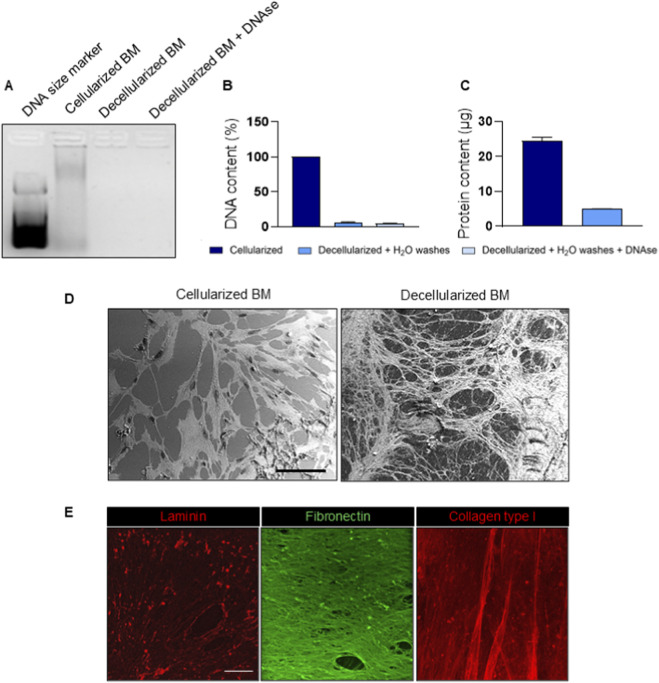
Characterization of cardiac biomatrix produced in vitro by AHCFs. **(A)** Agarose gel electrophoresis of cellularized and decellularized samples. Electrophoresis was performed at 150 V for 15 min in 1.5% agarose. Lane 1 – DNA size marker 100–3000 bp; 2 – cellularized BM; 3 – decellularized BM washed with water; 4 – decellularized BM washed with water and treated with DNAse. **(B)** Quantification of residual DNA after decellularization process through spectrophotometer. **(C)** BM protein content quantification using BCA colorimetric assay; cellularized sample was used as control. **(D)** Representative scanning electron microscopy images showing the architecture of the AHCF-deposited BM before and after decellularization. Scale bar = 100 µm. **(E)** Immunofluorescence images for laminin (red), fibronectin (green) and collagen type I (red) on decellularized BM samples. Scale bar = 100 µm.

In this study, a cardiac tissue-like ECM biomatrix (BM) was prepared by culturing AHCFs on polystyrene culture plates for 21 days, in accordance with previously published protocols (Paoletti et al., 2022; Castaldo et al., 2013). Upon decellularization and freeze-drying, a soluble powder was obtained. BM powder was stored at −20 °C and resuspended in ultrapure H_2_O before coating preparation. To collect high-quality BM samples, the decellularization protocol was optimized preventing protein denaturation and minimizing DNA residues. AHCFs were decellularized after 21 days culture and washed with ultrapure water, while non-decellularized AHCFs were used as control. Agarose gel electrophoresis was performed to detect any residual traces of nucleic acids. A defined band of nucleic acids was observed in the cellularized samples, while no signal was detected for any of the decellularized samples (Figure 1A). No relevant differences were observed between DNase-treated and untreated decellularized samples, indicating efficient DNA removal in both conditions. Spectrophotometric measurements confirmed the effective elimination of DNA even without DNase, showing negligible DNA percentages for all conditions, with no statistically significant differences (Figure 1B). Then, total protein content of the BM was determined. The cellularized samples contained approximately 25 μg/mL of total protein. In contrast, the decellularized samples, washed with distilled water, showed significantly lower protein content of 4.95 μg/mL (Figure 1C). The micro-architecture of BM was observed using SEM (Figure 1D). In not decellularized samples, SEM images showed the presence of an ECM covering the substrate, with elongated cells embedded within the deposited matrix. The surface was partially masked by cellular bodies and cytoplasmic extensions, forming a continuous layer over the underlying fibrillar structures. Following decellularization, cellular components were no longer detectable. BM samples displayed a fibrillar architecture organized in an interconnected network of thin fibers distributed across the surface. The matrix exhibited a porous structure with irregularly shaped pores of variable size, delineated by densely packed fibrillar bundles. The overall morphology of the BM appeared preserved after decellularization, with no evident disruption of the fibrillar network. Then, protein composition of BM post-decellularization process was investigated by immunofluorescence. BM was positively stained for laminin, fibronectin and collagen type I, while no nuclei signals were detected (Figure 1E). Fibronectin and laminin displayed a well-defined network, while collagen I had a less organized structure.

### 3.2 Influence of ECM protein coatings on miRcombo-mediated direct reprogramming of AHCFs into iCMs

The effect of different ECM protein coatings on miRcombo-mediated direct reprogramming of AHCFs was evaluated. After 24 h of transient transfection with DE-DOPE/miRcombo lipoplexes, cells were detached and seeded onto different culture substrates with no coating (NC), or coated with either laminin (LN), fibronectin (FN), collagen I (CI) or biomatrix (BM). Reprogramming efficiency was analysed at 7 and 15-day post cell culture on protein coatings, following experimental timeline shown in Supplementary Figure S1.

**FIGURE 2:**
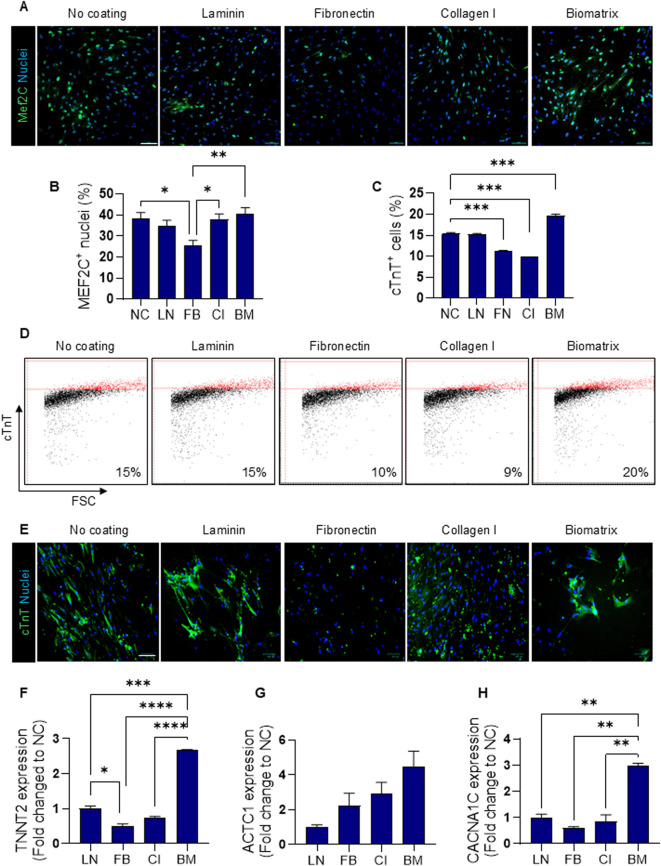
Fibroblast reprogramming via DE-DOPE/miRcombo lipoplexes is influenced by cardiac ECM protein coatings. **(a,b)** Immunofluorescence images **(a)** and quantification **(b)** for Mef2C (green) in AHCFs transfected with DE-DOPE/miRcombo lipoplexes and cultured for 7 days on NC and LN, FN, CI and BM coated plates. Scale bar = 100 µm. Percentage of Mef2C positive cells was calculated by counting positive nuclei for Mef2C on total nuclei number; **(c,d)** Flow cytometry plots **(c)** and quantification **(d)** for cTnT-positive AHCFs transfected with DE-DOPE/miRcombo lipoplexes, after 15 days culture on NC and LN, FN, CI and BM coated plates; cTnT positive cells are shown in red; **(e)** Immunofluorescence images for cTnT (green) expression in cells transfected with DE-DOPE/miRcombo lipoplexes, after 15 days culture on NC and LN, FN, CI and BM coated plates. Scale bar = 100 µm. **(f–h)** Gene expression analysis for TNNT2 **(f)**, ACTC1 **(g)** and CACNA1C **(h)** using ddPCR on cells transfected with DE-DOPE/miRcombo lipoplexes, after 15 days culture on NC and LN, FN, CI and BM coated plates. Data are expressed as fold change relative to the NC condition. Data are expressed as mean ± SEM of three independent experiments. Statistical analysis was performed by 1-way ANOVA.

**FIGURE 3:**
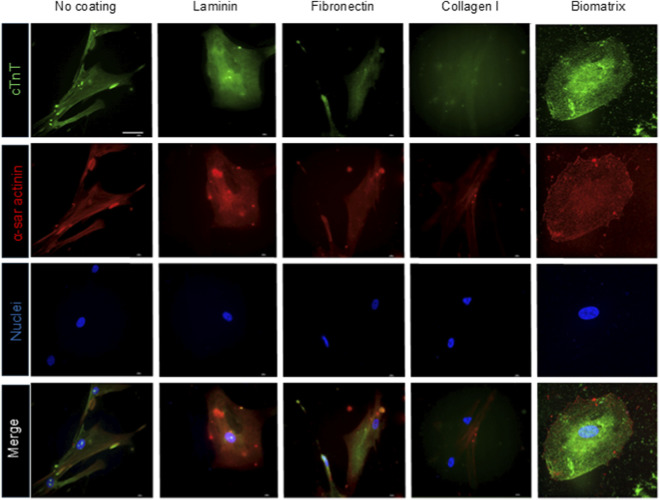
The expression of structural cardiac proteins is influenced by ECM protein coatings. Immunofluorescence for cTnT (green) and α-sarcomeric actinin (red) proteins in AHCFs transfected with DE-DOPE/miRcombo lipoplexes cultured for 15 days on non-coated, laminin, fibronectin, collagen I and biomatrix-coated plates. Scale bar = 50 µm. Nuclei were counterstained with Dapi (blue).

Immunofluorescence for Mef2C transcription factor was assessed at 7-day cell culture on protein coatings. AHCFs exhibited nuclear Mef2C expression under all culture conditions (Figure 2A). Quantification analysis revealed comparable percentages of Mef2C^+^ nuclei (∼35–40%) for NC, LN, CI, and BM conditions while a significantly lower percentage (∼20%) was measured using FN coatings (*p < 0.05; *p < 0.01; Figure 2B). Reprogramming efficiency of AHCFs treated with DE-DOPE/miRcombo lipoplexes was further investigated by flow cytometry after 15 days culture on protein coatings by analysing cTnT expression. The percentage of cTnT^+^ cells was ∼15% on NC and LN coatings, ∼20% on BM and ∼10% on FN and CI coatings (Figures 2C,D). Immunofluorescence images for cTnT (Figure 2E) showed that cells cultured on NC, and LN and BM coatings showed a more functional arrangement of cTnT in the cytoskeletal compartment compared to cells cultured on FN and CI coatings, which exhibited very low expression of cTnT with no evident cytoplasmatic expression. Gene expression of TNNT2 (encoding for cTnT), CACNA1C (calcium voltage-gated channel subunit alpha-1C), and ACTC1 (encoding for actin alpha cardiac muscle 1) was evaluated by ddPCR after 15 days of culture on protein coatings. Droplet digital PCR analysis revealed that coatings differently influenced the upregulation of cardiac markers upon miRcombo-mediated reprogramming (Figures 2F–H). TNNT2 expression was higher for cells cultured on LN, CI and BM compared to FN coatings (*p < 0.05, ***p < 0.001, ***p < 0.0001, respectively), with the highest fold change observed on BM coatings (Figure 2F). ACTC1 and CACNA1C expression levels progressively increased for cells cultured from LN to CI and BM coatings, with BM yielding a significant upregulation of all three cardiac genes (**p < 0.01 vs. LN, FB, and CI; Figures 2G,H). High-magnification immunofluorescence images were obtained by staining cTnT and α-sarcomeric actinin (α-SAR) (Figure 3). Immunofluorescence images evidenced the expression of cTnT and α-SAR cardiac proteins in NC, LN and BM. In contrast, cells cultured on FN and CI showed very low cTnT and α-SAR signal, highlighting the effect of ECM protein coatings in DE-DOPE/miRcombo lipoplexes alone on cell reprogramming.

### 3.3 YAP signaling controls direct fibroblast reprogramming on different ECM protein coatings

**FIGURE 4:**
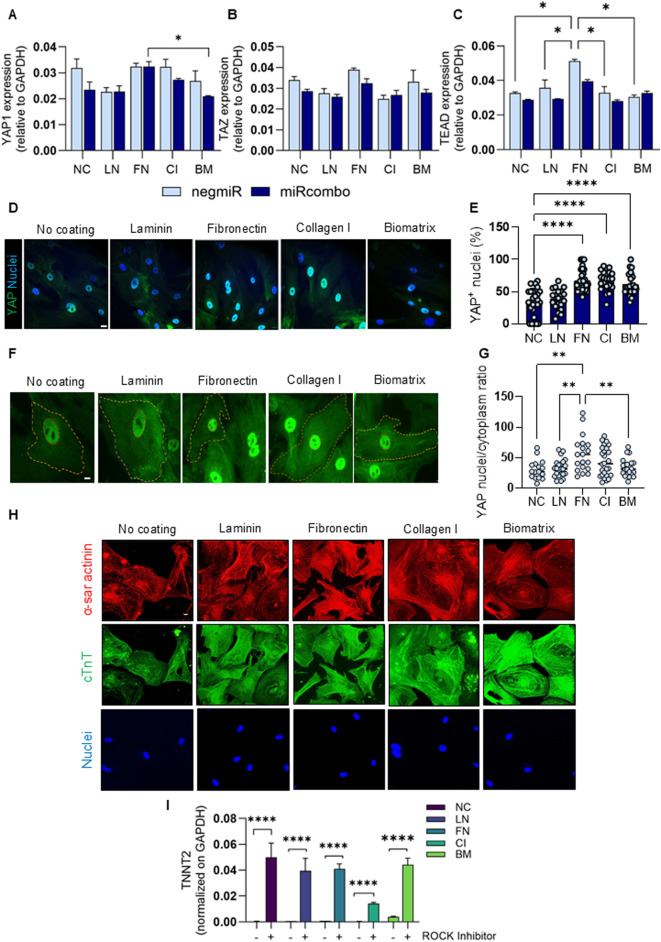
Protein coating-dependent regulation of Ki67 proliferation marker and YAP activation for AHCFs transfected with DE-DOPE/miRcombo lipoplexes. **(A–C)** Gene expression analysis for YAP1 **(A)**, TAZ **(B)** and TEAD **(C)** using ddPCR on AHCFs, transfected with negmiR or miRcombo using DE-DOPE lipoplexes, after 3 days of culture on NC and LN, FN, CI and BM coated plates. Data are expressed as mean ± SEM of three independent experiments. Statistical analysis was performed by 1-way ANOVA. **(D,E)** Immunofluorescence images **(D)** and quantification **(E)** for YAP protein (green) in AHCFs, transfected with DE-DOPE/miRcombo lipoplexes, cultured for 3 days on NC, LN, FN, CI and BM-coated plates. Scale bar = 20 µm. Percentage of YAP positive cells was calculated by counting positive nuclei for YAP on total nuclei number. **(F)** Representative high-magnification images showing YAP subcellular localization in AHCFs transfected with DE-DOPE/miRcombo lipoplexes, cultured for 3 days on NC, LN, FN, CI and BM-coated plates. Nuclear and cytoplasmic regions were manually defined based on YAP fluorescence signal. Scale bar = 10 µm. **(G)** Quantification of YAP nuclear-to-cytoplasmic (N/C) fluorescence intensity ratio calculated from images in **(F)**. For each cell, mean fluorescence intensities were measured within nuclear and cytoplasmic ROIs after background subtraction. **(H)** Immunofluorescence for cTnT (green) and α-sarcomeric actinin (red) proteins in AHCFs transfected with DE-DOPE/miRcombo lipoplexes and treated with ROCK Inhibitor for 15 days on non-coated, laminin, fibronectin, collagen I and biomatrix-coated plates. Scale bar = 10 µm. Nuclei were counterstained with Dapi (blue). **(I)** Gene expression analysis for TNNT2 using ddPCR on AHCFs, transfected with miRcombo using DE-DOPE lipoplexes with or without ROCK-Inhibitor, after 15 days of culture on NC and LN, FN, CI and BM coated plates. Data are expressed as mean ± SEM of three independent experiments. Statistical analysis was performed by 1-way ANOVA.

Given the established role of YAP signalling as a mechanotransducer integrating environmental cues and cell fate regulation, we next investigated whether cell proliferation and YAP signaling of AHCFs treated with DE-DOPE/miRcombo lipoplexes are influenced by the protein coatings. Firstly, proliferation was analysed in AHCFs (after transfection with DE-DOPE/miRcombo lipoplexes), seeded onto the different protein coated plates. After 3 days, cell proliferation was analysed by investigating the expression of Ki67 protein, a well-established proliferation marker. LN induced very low proliferative activity (<3% Ki67^+^ cells), whereas the highest proliferation rate was reached on FN (∼17%) and CI (∼11%) (Supplementary Figure S2). FN triggered a percentage of Ki67^+^ positive cells about six times higher than LN (****p < 0.0001). No statistical differences were detected among NC, CI and BM coatings. Since YAP signaling acts as a mechanosensitive regulator, linking ECM cues to cell proliferation and reprogramming, the expression of YAP1, TAZ, and TEAD genes was investigated by ddPCR. As shown in Figure 4A, YAP1 expression was significantly higher in cells cultured on FN coatings, both negmiR and miRcombo conditions, while the lowest expression levels were consistently detected in cells cultured on LN-coated plates, both in negmiR- and miRcombo-transfected cells. For CI and BM coatings, YAP1 levels were higher in negmiR-transfected cells, whereas miRcombo transfection resulted in a marked downregulation of YAP1 expression. No significant differences were observed in TAZ expression among the different ECM coatings, except for a slight decrease in cells cultured on BM under negmiR conditions (Figure 4B). TEAD expression showed a significantly higher expression in negmiR-transfected cells cultured on FN coatings compared to NC and all others ECM protein coatings (Figure 4C). Immunofluorescence staining showed distinct YAP subcellular localization patterns depending on the protein coating (Figure 4F). A stronger nuclear YAP signal was observed in cells cultured on FN and CI coatings, whereas the fluorescence intensity appeared weaker on LN- and BM-coated as well as NC plates. Quantification confirmed a significantly higher percentage of YAP^+^ nuclei for cells cultured on FN and CI coatings compared to NC and LN-coated plates (****p < 0.0001), with cells cultured on BM coatings showing intermediate values (Figure 4G). High-magnification images further highlighted the nuclear enrichment of YAP under these conditions (Figure 4H). Consistently, the nuclear-to-cytoplasmic (N/C) YAP intensity ratio was significantly higher for cells cultured on FN-coated plates compared with LN- and BM-coated ones and NC condition (**p < 0.01; Figure 4I). A higher N/C ratio indicates predominant nuclear localization of YAP, reflecting its activation state and supporting enhanced YAP signaling for cells cultured on FN coatings and, to a lesser extent, on CI coatings. Next, to determine the role of YAP in driving ECM coatings and miRcombo mediated reprogramming, AHCFs transfected with miRcombo using DE-DOPE were treated with Y-27632, a well-established ROCK Inhibitor which is frequently used also to downregulate YAP signalling (Kurotsu et al., 2020). After 15 days, cells cultured in NC or in different ECM protein coatings clearly displayed cTnT and α-sarcomeric actinin (Figure 4H) and significantly higher expression of TNNT2 mRNA compared to non-treated cells (Figure 4I).

## 4 Discussion

Although direct reprogramming has been widely exploited to target and revert cardiac scarring in animal-based models, translating this approach to adult human cells still remains a challenge due to the low efficiency of this phenotype switch and the limited maturation level of iCMs (Vaseghi et al., 2017). Previously we designed DE-DOPE/miRNA lipoplexes able to efficiently and safely transfect AHCFs *in vitro*, resulting in enhanced *in vitro* direct reprogramming efficiency of AHCFs into iCMs compared to commercial DharmaFECT1 lipoplexes (Nicoletti et al., 2022). On the other hand, the effect of microenvironmental biochemical and biophysical cues on *in vitro* direct reprogramming efficiency and iCMs maturation (Paoletti et al., 2022; Vaseghi et al., 2017). Specifically, the effect of ECM-mimetic substrates on direct cardiac reprogramming of mouse fibroblasts into iCMs was previously investigated by seeding fibroblasts treated with a combination of drugs onto laminin and RGD-functionalized PEG hydrogels (Smith et al., 2013). Additionally, Matrigel was exploited either as a coating for polyacrylamide hydrogels or as an additive in fibrin hydrogels to study the reprogramming efficiency of mouse fibroblasts into iCMs (Li Y. et al., 2016). However, Matrigel, a reconstituted basement membrane rich in laminin and collagen IV, is derived from Engelbreth–Holm–Swarm mouse tumours, presents significative lot-to-lot variability, and does not accurately replicate the physiological microenvironment of human cardiac tissue (Aisenbrey and Murphy, 2020; Oberwallner et al., 2015). Additionally, its animal-tumour origin does not comply with the 3Rs (Replacement, Reduction, and Refinement) principle (Duarte et al., 2023).

Starting from these early studies and their limitations, herein we explored the effect of different coatings based on individual ECM proteins versus *in vitro* produced cardiac BM on the behavior of AHCFs transfected with DE-DOPE/miRcombo lipoplexes, in terms of proliferation, direct reprogramming efficiency and maturation of iCMs. Specifically, BM was produced *in vitro* by culturing AHCFs for 21 days. Indeed, cardiac fibroblasts actively synthesize and secrete ECM proteins, which progressively accumulate on the culture dish surface (Nurzynska et al., 2024; Chacar et al., 2017). Following the optimized decellularization protocol, AHCF-derived BM displayed an interconnected network of thin fibrillar elements that remained structurally preserved (Figure 1D). Consistently, immunofluorescence analysis confirmed the retention of key structural and adhesive ECM components, including laminin, fibronectin, and collagen type I, yielding a cardiac-like ECM composition (Figure 1E) (Silva et al., 2021).The preservation of these proteins is crucial, as they provide essential cues that guide cell adhesion, cytoskeletal remodeling, and lineage specification (Li et al., 2018)*. In vitro* produced BM showed a well-defined fibrillar structure for laminin and fibronectin, while collagen I appeared not completely organized and crosslinked. This was probably due to the physiological phenotype of AHCFs responsible for BM deposition; indeed, Castaldo *et al.* reported that organized collagen I deposition and its upregulation occur in pathological BM produced by myofibroblasts (Castaldo et al., 2013). Moreover, decellularization process removed cellular debris and nucleic acids (Figures 1A,B). The complete removal of DNA is an essential requirement to avoid cytocompatibility issues and prevent immune reactions (Chacar et al., 2017). These findings confirmed the feasibility of generating an *in vitro*-produced human BM that closely mimics native cardiac ECM composition, thus offering a species- and tissue-specific substrate for reprogramming studies. In this work, the role of single and pooled- ECM proteins has been investigated in modulating miRcombo-mediated direct reprogramming of AHCFs into iCMs. Reprogramming efficiency, assessed mainly by Mef2C and cTnT expression, varied significantly for miRcombo-transfected cells cultured on the preotein coated plates. Nuclear localization of Mef2C across all coatings confirmed the induction of iCM-like phenotype (Figures 2A,B). Since Mef2C works synergistically with other cardiac transcription factors (Gata4, Tbx5 and Hand2) in directing cardiac cell fate and contractile protein expression (Isomi et al., 2021; Akazawa and Komuro, 2003; Desjardins and Naya, 2016), its nuclear localization provides a critical indicator of successful transcriptional reprogramming. Its expression was slightly higher for cells cultured on laminin and BM coatings, suggesting that these substrates better support cardiac lineage initiation. In contrast, cells cultured on fibronectin-coated surfaces showed reduced Mef2C^+^ nuclei, indicating a less favorable microenvironment for cell reprogramming. The phenotype switch from AHCFs to iCMs was further evaluated by focusing on cardiac Troponin T, as part of the Troponin complex, a key protein involved in cardiac muscle contractility (de A Marques, 2016). A higher percentage of cTnT^+^ cells was detected on BM compared to single protein coatings (Figures 2C,D). Laminin also supported cardiac phenotype induction, however without statistically significant differences compared to non-coated plates. On the other hand, a significantly lower percentage of cTnT^+^ cells was present on fibronectin and collagen I coatings, suggesting that they both decrease direct reprogramming efficiency. Flow cytometry results were confirmed by immunofluorescence analysis for cTnT (Figure 2E). Indeed, miRcombo-transfected cells cultured on fibronectin and collagen I coatings displayed very low cytoplasmatic expression of cTnT protein. Conversely, laminin and biomatrix coatings enhanced cTnT expression of miRcombo-transfected cells. Gene expression analysis further confirmed the influence of coating composition on reprogramming efficiency (Figures 2F–H). MiRcombo-transfected cells cultured on BM showed a significant upregulation of TNNT2, ACTC1, and CACNA1C, which suggested higher activation of cardiac structural and functional genes. Laminin coatings also supported increased TNNT2 expression in miRcombo-transfected cells. On the contrary, cells cultured on fibronectin and collagen I coatings showed markedly lower levels of all cardiac markers, suggesting they support a fibroblast-like transcriptional profile less permissive to cardiac conversion.

These findings are in agreement with previous studies highlighting the role of laminin- and Matrigel rich substrates in enhancing cardiac conversion compared to control uncoated culture plates (Zhang et al., 2012; Kurotsu et al., 2022; Wang et al., 2019). Indeed, laminin binds integrin β1 and dystroglycan receptors that promote cardiogenic signaling cascades and the organization of contractile proteins (Casarella et al., 2025; Li S. et al., 2016). Conversely, fibronectin and collagen I are abundant in fibrotic tissue and are known to maintain fibroblast identity through integrin-mediated mechanosensing pathways (Herum et al., 2017; Ieda et al., 2009).

The expression of cardiomyocyte proteins by miRcombo-transfected cells cultured on the different coatings confirmed their role on direct cardiac reprogramming. Specifically, cells cultured on laminin or BM exhibited cTnT and α-sarcomeric actinin (Figure 3). In contrast, miRcombo-transfected cells cultured on fibronectin and collagen I showed spotted expression of cardiac markers, suggesting limited functional maturation. These differences likely arose from substrate-specific modulation of cell–matrix adhesion and traction forces, which in turn affected intracellular signaling and gene activation (Dupont, 2016). We next explored the molecular mechanisms through which these extracellular cues influence fibroblast-to-cardiomyocyte conversion. We observed that miRcombo-transfected fibroblasts showed different proliferation rate when cultured on ECM protein and BM coatings. This difference was observed since the first 72 h culture time, suggesting that cell–substrate adhesion was transduced into transcriptional pathways controlling proliferation and differentiation. Indeed, fibronectin and collagen I coatings induced higher proliferation rates than laminin and BM coatings, as evidenced by the higher Ki67^+^ cell percentages (Supplementary Figure S2). These findings are consistent with previous studies showing that ECM proteins mediate cell communication by regulating several biological processes, although none has demonstrated a direct link between direct cell reprogramming and proliferation rate. For example, Valiente *et al.* explored the implications of ECM-cell interactions in myocardial cell behaviour and cardiac function. They have reported that FN and CI can induce cell spreading, migration and proliferation (Valiente-Alandi et al., 2016). Moreover, Ieda *et al.* have demonstrated that these two proteins can stimulate CMs proliferation by interacting with β1 integrin (Ieda et al., 2009). Previous studies have also demonstrated that laminin can initiate and drive differentiation into CMs (Yap et al., 2019).

We then explored whether the proliferation rate of cells cultured on the different protein and BM coatings could be due to a different YAP signaling activation. Indeed, YAP protein belongs to the Hippo pathway, acts as an mechanosensory component of cells, transducing biophysical cues at the membrane interface and regulating response via controlling differentiation and proliferation in cells (Fu et al., 2022). YAP and its co-factor TAZ are the main downstream effectors of the Hippo pathway, exerting their activity by binding to TEAD transcriptional factors (Cunningham and Hansen, 2022). YAP activation, and thus its nuclear translocation, is related to several biological processes including organ growth, tissue fibrosis, tissue renewal and cell proliferation (Piccolo et al., 2014). In cardiac fibroblasts, YAP becomes activated after myocardial infarction, promoting proliferation, myofibroblast differentiation, and the expression of ECM-associated genes (Piccolo et al., 2014). Activation of YAP enhances the reprogramming efficiency of somatic cells into induced pluripotent stem cells (iPSCs) (Di Benedetto et al., 2021; Kurotsu et al., 2020; Camargo et al., 2007; Qin et al., 2012). Conversely, the suppression of YAP/TAZ signalling and the silencing of fibroblast gene programs facilitates cardiac progenitor cells transition from the proliferative to the differentiation stage (Mochizuki et al., 2017). In the field of direct reprogramming, increased reprogramming efficiency has been shown to be inversely correlated with YAP/TAZ activity. This effect was observed when cells were cultured on soft matrices that more closely recapitulate the mechanical stiffness of the native cardiac microenvironment (Roy et al., 2025; Kurotsu et al., 2020). To gain mechanistic insight, we investigated YAP signaling activation in miRcombo-transfected AHCFs cultured on different ECM protein coatings. We found that fibronectin and collagen I coatings promoted elevated YAP1 expression (Figure 4A) and nuclear YAP localization (Figures 4D,E), consistent with a pro-fibrotic microenvironment that sustains cell proliferation. Conversely, laminin and BM coatings suppressed YAP nuclear translocation, favoring cardiomyocyte differentiation (Figures 4D,E). Notably, these differences were not limited to YAP subcellular localization but were also reflected in marked variations in fluorescence intensity (Figures 4F,G). Fibronectin, but also collagen I, coated substrates showed a stronger YAP signal compared to laminin and BM conditions, indicating higher overall YAP abundance and activity. In contrast, laminin and BM coatings were associated with reduced YAP fluorescence intensity, consistent with attenuated YAP signaling.

To further assess the functional role of YAP in this context, we treated miRcombo-transfected AHCFs with a ROCK inhibitor, a known modulator of cytoskeletal tension that also suppresses YAP activity. Remarkably, ROCK inhibition led to a substantial improvement in reprogramming efficiency, highlighting that YAP suppression is a critical determinant in promoting the transition from fibroblast to cardiomyocyte-like phenotypes (Figures 4H,I). This observation aligns with prior reports showing that decreased YAP/TAZ activity facilitates direct cardiac reprogramming, particularly in soft or biomimetic environments that resemble native cardiac stiffness (Kurotsu et al., 2020). These results suggest that YAP inhibition may be necessary to achieve full reprogramming as previously suggested, confirming the role of the ECM microenvironment on direct cardiac reprogramming (Paoletti et al., 2022; Kurotsu et al., 2020). While miRcombo directly targets transcriptional networks to activate cardiac genes (Jayawardena et al., 2012; Paoletti et al., 2020), ECM proteins determine how effectively these programs are executed by controlling cell adhesion, cytoskeletal organization, and mechanosensitive transcription factors such as YAP. This interplay might explain why reprogramming efficiency and maturation are higher *in vivo* than in conventional 2D cultures, where cells experience non-physiological stiffness and lack cardiac-specific ECM cues. In this context, biomimetic substrates, such as BM, provide an instructive environment that recapitulates the biochemical composition and mechanical stiffness of the native myocardium, thereby improving reprogramming efficiency and promoting structural organization of iCMs. Taken together, our findings indicate that the variations in cellular proliferation are likely driven by differences in how cells undergoing reprogramming interact with specific extracellular matrix proteins. Distinct ECM proteins appear to modulate adhesion-mediated signaling pathways, influencing both YAP activity and downstream proliferative responses. These observations are consistent with evidence showing that ECM composition, rather than mechanical cues alone, can play a decisive role in regulating the efficiency of direct reprogramming by shaping cytoskeletal organization, transcriptional dynamics, and lineage commitment. Overall, this study highlights the crucial role of ECM–cell interactions in the direct reprogramming process, suggesting that tailoring the composition of the culture substrate could be a promising strategy to enhance reprogramming outcomes.

## 5 Conclusion

This study demonstrates that ECM protein composition significantly influences miRcombo-mediated direct reprogramming of AHCFs by modulating cell behavior, cytoskeletal organization, and mechanosensitive signaling pathways. Among the tested substrates, LN and BM coatings provided the most supportive microenvironment for cardiac marker expression and structural maturation, whereas fibronectin and collagen I were associated with lower reprogramming efficiency and a more proliferative phenotype. Our data indicate that differences in reprogramming outcomes may be associated with distinct patterns of YAP signaling and cell proliferation across ECM conditions. Substrates that supported higher reprogramming efficiency generally showed reduced YAP nuclear localization and lower proliferative activity at early time points, although this relationship was not strictly proportional across all conditions, suggesting that YAP modulation contributes to, but does not solely determine, reprogramming efficiency. Overall, these findings highlight that ECM biochemical cues play an instructive role in direct cardiac reprogramming by shaping adhesion-mediated and mechanotransduction responses. Tailoring substrate composition using laminin-enriched matrices represents a promising strategy to improve *in vitro* reprogramming performance and phenotypic maturation. Future approaches based on laminin-rich biomimetic matrices combined with three-dimensional microenvironments and physiologically relevant stiffness may further enhance reprogramming efficiency and functional maturation by more closely recapitulating native cardiac tissue conditions.

## Data Availability

The data presented in the study are deposited in the Zenodo repository, accession number https://doi.org/10.5281/zenodo.18847388
