# Supplementary material for: ECM proteins regulate microRNA-mediated direct reprogramming of fibroblasts into cardiomyocytes via YAP signaling
Source: Front Bioeng Biotechnol. 2026 Mar 12;14:1749865. doi: 10.3389/fbioe.2026.1749865 (PMC13018108; doi:10.3389/fbioe.2026.1749865)
Supplement: Supplementary file 1 [file Supplementaryfile1.docx]

Supplementary Material


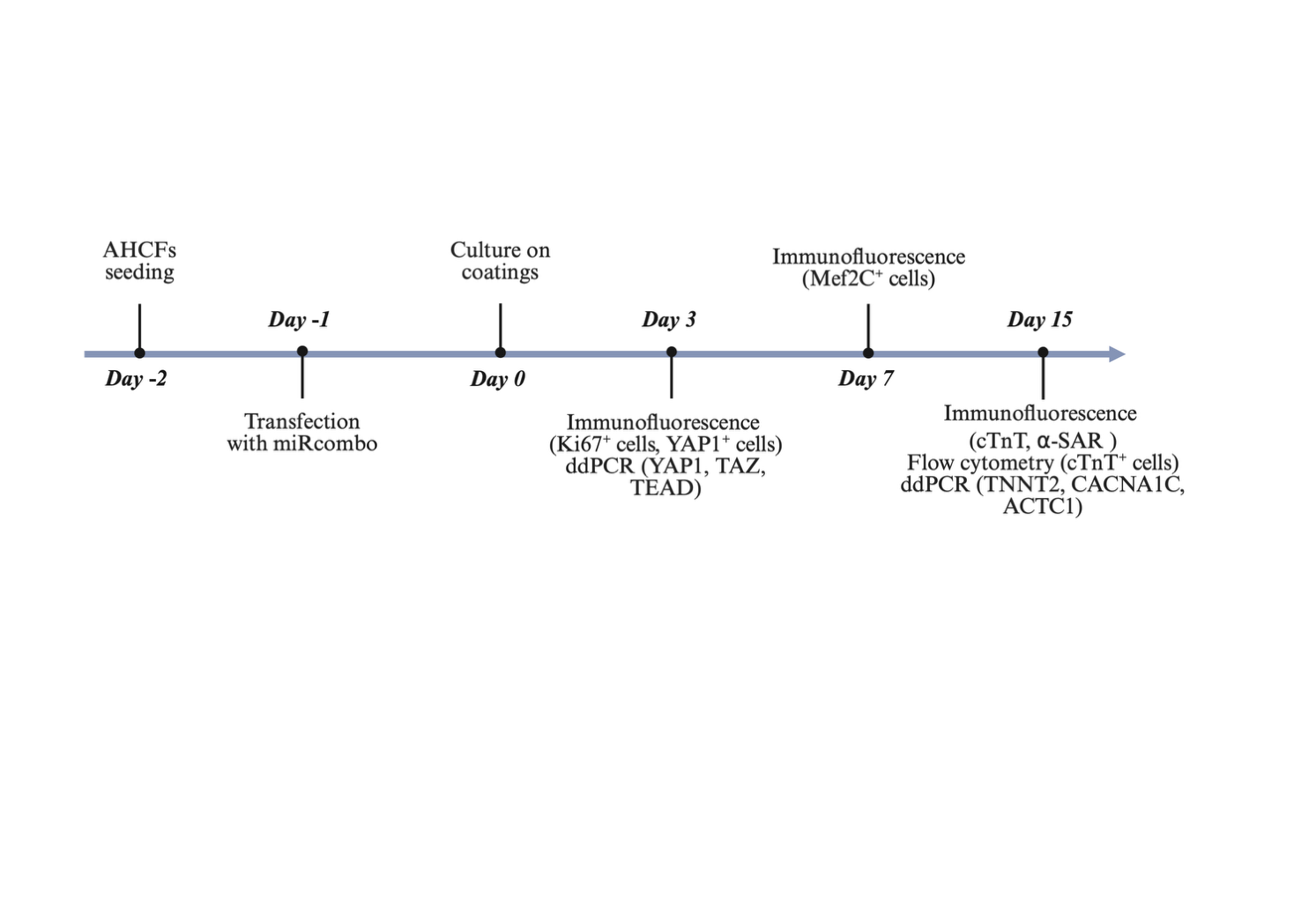


**Supplementary Figure 1.** Experimental timeline.


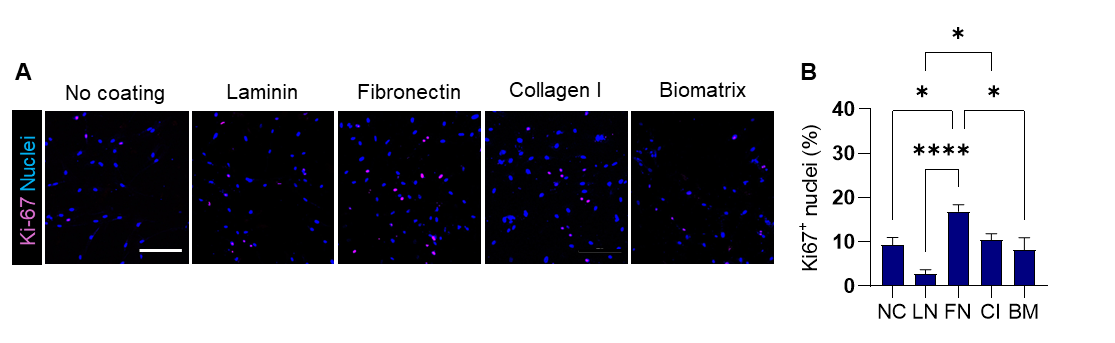


**Supplementary Figure 2: A,B)** Immunofluorescence images **(A)** and quantification **(B)** for Ki67 protein (magenta) in AHCFs, transfected with DE-DOPE/miRcombo lipoplexes, cultured for 3 days on NC and LN, FN, CI and BM coated plates. Scale bar = 250 µm. Percentage of Ki67 positive cells was calculated by counting positive nuclei for Ki67 on total nuclei number. Nuclei were counterstained with Dapi (blue).
